# Health professionals’ experiences with participating in quality improvement in elderly care

**DOI:** 10.1108/IJHCQA-11-2024-0103

**Published:** 2025-05-09

**Authors:** Lisabet Wieslander, Marie Haggström, Ingela Bäckström

**Affiliations:** Department of Communication, Quality Management and Information Systems, Mid Sweden University, Sundsvall, Sweden; Vård och Omsorgsförvaltningen, Sundsvall, Sweden; Department of Health Sciences, Mid Sweden University – Sundsvall Campus, Sundsvall, Sweden; Department of Communication, Quality Management and Information Systems, Mid Sweden University Campus Ostersund, Ostersund, Sweden

**Keywords:** Organizational improvement, Employee participation, Nursing homes, Organizational barriers, Quality of care, Work environment, Total quality management

## Abstract

**Purpose:**

Employee participation in quality improvement is essential for fostering engagement and job satisfaction and delivering high-quality care, as highlighted in the total quality management literature. However, how employee participation is facilitated during quality improvement processes within healthcare organizations remains largely unexplored. Previous studies have identified a research gap, highlighting the importance of examining this phenomenon from the perspective of health professionals. The purpose of this study was to gain a deeper understanding of how health professionals in nursing homes experience their participation in quality improvement. The underlying aim was to describe their involvement and how it can be strengthened, ultimately improving the quality of care.

**Design/methodology/approach:**

This study is based on interviews with 17 health professionals from 2 nursing homes in a medium-sized Swedish municipality. Data were analysed with Reflexive Thematic Analysis.

**Findings:**

The themes identified through the analysis are “experiences of barriers at the organizational level that limit participation in quality improvement” and “experiences of barriers in daily care that limit participation in quality improvement”.

**Originality/value:**

The themes address barriers to participation in quality improvement, reflecting an organizational structure that hinders participation and quality improvement. A quality culture, along with structured approaches to improve quality and foster employee participation is lacking. Implementing total quality management could help address the challenges faced by health professionals in these settings.

## Introduction

Improving the quality of care by implementing new research evidence and technology is crucial for healthcare organizations, not only to reduce medical errors and serious patient incidents but also to enhance the work environment for health professionals (Kostman and Sastry, 2019). Currently, recruiting and retaining staff is challenging, and this problem is expected to escalate in the coming years as the demand for elderly care services increases due to significant demographic shifts in society (European Commission, 2018). This represents one of the major societal challenges of our time, and policy-makers predict that implementing new technology and evidence will be essential for delivering high-quality care in the future (Leung *et al*., 2021). This represents a crucial area of research for exploring how quality improvement (QI), described in elderly care as a process of change involving implementing new evidence and technologies, can be strengthened and optimized to ensure that elderly care meets future challenges without compromising quality.

### Aim

The purpose of this study was to gain a deeper understanding of how health professionals in nursing homes experience their participation in quality improvements. The underlying aim was to describe their involvement and how it can be strengthened, ultimately improving the quality of care.

Research questions:

RQ1.
How do health professionals experience their participation in quality improvement?

RQ2.
What barriers exist to participating in quality improvement in elderly care?

RQ3.
How can the participation of health professionals in quality improvements improve the quality of care?

#### Nursing homes

Sweden’s 290 municipalities are responsible for healthcare under the Health and Medical Services Act (SFS, 2017) and for elderly care under the Social Services Act (SFS, 2001). Nursing homes provide both medical and social care. Registered nurses (RNs) oversee care, but most care is provided by nurse assistants, an increasing number of whom lack formal education (National Board of Health and Welfare, 2016). Despite efforts to deliver high-quality care, nursing home standards are often considered inadequate (Ersek and Carpenter, 2013).

#### Quality improvement

The Institute of Medicine defines quality of care as the degree to which health services improve outcomes and align with professional knowledge (IOM, 1990). However, broad definitions can be challenging to apply, leading to the use of quality indicators. The fundamental premise of QI is that a well-structured, organization-wide strategy enhances work processes, ultimately leading to better outcomes (Donabedian, 1985). However, previous research suggests that simply having access to outcome data does not guarantee successful QI implementation (Zuidgeest *et al*., 2014). In nursing homes, various QI initiatives are commonly undertaken, including the development of individualized care plans, staff training, and the establishment of care guidelines (Winters *et al*., 2016). Despite these efforts, many QI activities have little direct impact on client outcomes, and in some cases, they may even have adverse effects (Werner *et al*., 2013). A deeper understanding of the mechanisms behind QI in elderly care is needed to achieve successful outcomes effectively.

#### Employee participation

Employee participation in QI has long been recognized as crucial for fostering engagement and motivation among staff (Klein *et al*., 2020). It has also been shown to play a significant role in employees’ decisions to remain within an organization, thereby reducing staff turnover (Zink, 2008). Since the inception of the quality management movement in the 1970s, many prominent researchers have highlighted the importance of participation in QI efforts (Hackman and Wageman, 1995). Employee participation is a fundamental concept in total quality management (TQM), emphasizing its importance in achieving higher service quality (Dahlgaard-Park *et al*., 2018). Fostering commitment from all employees and cultivating a culture of innovation, where management actively supports and values improvement initiatives from staff, are key elements in building a quality-driven organizational culture (Dahlgaard-Park *et al*., 2013). Giving staff decision-making autonomy can increase job satisfaction and reduce turnover (Chamberlain *et al*., 2016). However, how organizations actively promote this involvement and how it is perceived by employees are less explored. There is a knowledge gap, as few qualitative studies have examined how health professionals experience employee participation in QI in elderly care.

## Method

### Study design and sample

This study is based on a qualitative, inductive methodology with data from in-depth individual semi structured interviews with 17 health care professionals at 2 municipal nursing homes in a medium-sized municipality in northern Sweden (14 registered nurses, 1 physiotherapist, 1 occupational therapist, and 1 rehabilitation assistant). The inductive approach enables knowledge generation and understanding of new insights from the interviews (Patton, 2015). The investigation uses an exploratory methodology to examine and articulate the phenomenon of employee participation in QI.

One of the nursing homes employs approximately 100 nurse assistants and 6 nurses, whereas the other has approximately 140 nurse assistants and 10 nurses, along with rehabilitation professionals. All the study participants, except one, were women. The length of experience as health professionals ranged from 1 to 32 years, with a mean of 15 years of experience.

### Data collection

Individual interviews were conducted from February to April 2024. The interviews were conducted at the participants’ workplace in the nursing home in a closed room with no interruptions. The interviews lasted between 40 and 60 min and were recorded digitally. Probes and clarifying questions were used to encourage detailed responses, encompassing both positive and negative aspects to minimize bias. The research team then pseudonymized and transcribed the interviews verbatim.

### Data analysis

The qualitative data analysis began during the interviews and remained an ongoing process. Following each interview, the researchers dedicated time to reflect on the interview content, considering both spoken and unspoken aspects. Immediate reflections encompass emotions and sentiments expressed during the interviews, along with initial thoughts on potential emerging themes (Braun and Clarke, 2019). These initial reflections serve as valuable insights during the subsequent analysis stages. For coding, a research question-led, inductive approach was employed, encompassing both semantic and latent coding. The data were analysed via reflexive thematic analysis (RTA), an interpretative and theoretically flexible approach to identify themes within and across participants’ accounts in qualitative data. The six steps of RTA were used to assess the latent content of the data (Braun and Clarke, 2006, 2019, 2021).

Triangulation was employed to minimize researcher bias and enhance rigour. Codes and themes were developed through team discussions. Reflexive engagement was maintained through written reflections and monthly meetings. The software program NVivo was used for analysis, ensuring transparency.

### Ethics

The participants were informed about the study and provided written and verbal consent. Confidentiality and anonymity were strictly maintained. The study follows the ethical guidelines of the Swedish Research Council (2017), Mid Sweden University (2021), and the ICN Code of Ethics for Nurses (2021). Ethical considerations were integrated throughout the research process, with approval obtained from the relevant ethics committee in August 2023.

## Results

The analysis revealed two overarching themes that reflect health professionals’ experiences with QI: (1) “experiences of organizational barriers that limit participation in quality improvement” and (2) “experiences of barriers in daily care that limit professionals’ participation in quality improvement.” These main themes are further elaborated through subthemes (see Table 1).

**Table 1:** Thematic map presenting the overarching themes and subthemes

Theme	Subtheme
Experiences of organizational barriers that limit participation in quality improvement	Lack of organizational support hinders implementation of evidence and participation in quality improvement
	Initiative in quality improvement is stifled by top-down management, with financial organizational goals prioritized over care quality
	Restrictive organizational structures and unclear roles affect participation and teamwork in quality improvement
	Lack of participation and the digital burden throughout the organization hinder quality improvement
Experiences of barriers in daily care that limit participation in quality improvement	Unclear roles and distance from patient care hinder participation in quality improvements
	The structure of incident management hinders participation quality improvement
	Increased care complexity’s impact on care quality and participation in quality improvement

**Source(s):** Authors’ own work

### Experiences of organizational barriers that limit participation in quality improvement

This overarching theme encompasses four underlying themes related to how organizational structures create barriers to evidence-based practice (EBP) and participation in QI efforts. The hierarchical organizational structure, combined with unclear roles and responsibilities for quality improvement, poses significant challenges.

#### A lack of organizational support hinders the implementation of evidence and participation in quality improvement

Insufficient support for health professionals to work following EBP becomes a barrier to participation in QI. Professionals are left to their own EBPs but lack support for this effort, such as access to research databases to obtain the latest evidence and education on EBP. They also lack knowledge about what it means to work with QI using evidence. When asked if they actively work on implementing new evidence, one participant responded:

Well, we do work evidence-based, but it hasn’t really been anything new. So, no … -Interview 2

The examples of EBP that participants described in the interviews primarily originated from the field of medicine rather than from the research field specific to health professionals.

Yes, we do get some of that from the lunch lectures, where we receive updated information. But then again, you also need to have lunch sometimes. – Interview 4

Lunch lectures are held mostly by medical doctors, and health professionals attend them on their lunch breaks, which indicates a lack of support from the organization. New evidence is not systematically implemented or followed up within the organization. Health professionals expressed that new knowledge is not consistently disseminated; a process they find lacking. There is no structured time or effort dedicated to integrating evidence into daily care, which hinders both QI and the active participation of health professionals.

#### Top-down management stifles quality improvement initiatives, with financial and organizational goals prioritized over care quality

The nursing homes were perceived by health professionals as being governed from the top down, decisions about changes were made at higher levels, and they were often not informed about the rationale for these decisions. These decisions were sometimes seen as misaligned with the ethical responsibilities of health professionals, particularly with respect to care quality. The perception was that management was not working in their interest but rather pursuing its own agenda, focused on finances and budgeting rather than improving patient care.

… for example, the emergency medication storage, that we are not allowed to take medication during the day, but instead we must go to the pharmacy. And then we found out about this in a meeting, and we tried to argue that, well, there is actually healthcare, and we are supposed to provide good and safe care. But at the same time, we must go to the pharmacy to get medication when it is available in storage here. Interview 5

Perceived hierarchical leadership has resulted in a loss of motivation among health professionals to suggest improvements in care quality. They felt that their ideas went unheard, as previous development projects were stifled by an overriding focus on budget constraints and cost control.

No, I wouldn’t say it’s that easy to influence things; that’s not my experience. I can express what I think and feel, but I also sense that this isn’t going to lead anywhere. Sometimes, it has to go all the way to the top to those who make the decisions for anything to change.- Interview 1

The positive aspect of this theme was the strong commitment among health professionals to become more involved in QI efforts, even though they had partly stopped sharing ideas due to previous negative experiences when suggesting improvements.

… to sort of take the initiative for improvement, maybe it’s not a project, but I have many ideas, I don’t feel like I’m not allowed to start something that I’ve been thinking about … maybe something very small, just within the group itself, but … - Interview 12

Health professionals’ experiences with past improvement initiatives have focused primarily on organizational aspects rather than on care quality. At the same time, the perceived quality of care remains low, yet no systematic QI efforts have been implemented to address this. Health professionals generally believe that change management is lacking. Some managers were perceived to be more inclined towards change and more positive about improvement efforts than others. However, health professionals lacked direct examples of quality improvement initiatives specifically aimed at enhancing care quality within their wards, and there were instances where managers with clinical expertise were absent.

Within elderly care, good managers are needed. The most important thing is having good managers, you cant run any facility without having a good manager. And the manager must know what they are working with, it’s like if I were to work at Volvo as a manager. I’m really good with computers and such, but I don’t know anything about cars. That doesn’t work. Those who are managers must at least know a bit about what it’s like to work in elderly care. – Interview 11

While there were efforts to enhance care for individual patients—such as discussions in team meetings—there was a lack of comprehensive QI initiatives to improve overall care, particularly involving all health professionals in multidisciplinary team efforts. Health professionals did not have an answer on how a more systematic QI approach could be implemented. They described educational initiatives, but new knowledge of this education was often not seen in the everyday work on the wards. Ideas for improving care quality exist, but they are not allowed to be tested or implemented. Health professionals expressed a desire for greater involvement in initiatives aimed at improving care quality, such as strengthening the role of representatives and providing staff training. However, there was uncertainty about who holds responsibility for QI because of the organization’s division between social care (SoL) and health care (HSL).

#### Restrictive organizational structures and unclear roles affect participation and teamwork in quality improvement

Without effective teamwork between unit managers, care staff, health professionals, and management, QI efforts were difficult to initiate. Currently, there are challenges related to both organizational structure and lack of competence. Structures for how QI should be integrated into daily operations and who should be involved were missing. The approach for quality assurance in nursing homes uses representatives from care staff; however, without cross-professional teamwork in improvement efforts, the impact of these efforts remains limited.

I think the biggest challenge for all of us health professionals here is to get the care staff on board, to get them, like, we end up constantly having to push things forwards. It’s not something that happens naturally or on its own, and we always have to, and of course, that’s part of our role, but it’s a lot of having to be there and support things. – Interview 16

Health professionals felt that a fear of making mistakes, coupled with insufficient skills and inadequate training among care staff, contributed to a culture where staff hesitated to speak up when they did not fully understand instructions or prescriptions from health professionals. Teamwork was described as the most challenging aspect for health professionals. Informal leadership often emerges, leading to situations where instructions and directives from both health professionals and managers are questioned by nurse assistants.

It’s largely about defending and justifying why actions should be taken. It becomes a lot of subjective opinions and thoughts when there’s a lack of understanding, making it difficult to implement actions. It just becomes a burden. And then it doesn’t get done. It’s really challenging to be the person responsible for care in that situation. – Interview 6.

The high turnover rate among care staff further complicated efforts to establish effective teamwork, as it contributed to a fragmented environment with multiple regulations, managers, and silos between different parts of the organization, including the SoL and HSL. Moreover, an ethically complex care setting in which patients and their families suffer from inadequate care quality was described. All professions faced time constraints, as the organization had been streamlined for tighter budgets for several years. Additionally, there was no established protocol for team collaboration or a clear process for capturing and driving QI initiatives. Health professionals felt they were working in teams but were uncertain whether the care staff shared that perception.

#### Lack of participation and digital burdens throughout organizations hinder quality improvement

Implementing new technology in nursing homes was not based on the needs of healthcare staff. According to health professionals, involving staff in the process creates the conditions for successful and organization-adapted implementation. However, there was a general lack of effective technology implementation. Previous implementations of new technologies, such as electronic health record systems, faced significant challenges, leading to a decline in employees’ confidence in the success of future initiatives. The lack of participation poses a significant obstacle, as health professionals feel they are either unwilling or ill-equipped to embrace changes due to top-down decision-making, insufficient training, and limited opportunities for participating in the implementation process.

It feels like we’re working in a system designed for the primary healthcare centre. We’re supposed to schedule meetings, then carry them out, and eventually, there’s a result. It feels a bit like we’re moving away from actual care because so much administrative time is required just to complete a task. – Interview 4

Despite these challenges, health professionals expressed a strong desire to be more involved, and overall, their attitude towards new technology was positive. It was considered essential for delivering high-quality care, even though health professionals did not believe that technology would reduce the need for staff in elder care settings, particularly for dementia patients who require close, hands-on care to ensure their safety. Health professionals also emphasized the importance of ensuring that new technology is evidence-based and thoroughly tested. They highlighted the necessity of ethical reflections among practitioners to guarantee that technology provides safe, high-quality services that benefit both the older patients and the staff.

One example highlights how active staff participation and decision-making led to the effective implementation of new welfare technology. However, when management later decided to relocate the technology, the benefits for patients and the implementation’s value were lost.

I advocated for bringing it here, it was purchased, and it’s been used a lot. It’s a bit of new technology, a new way of thinking … the staff became so engaged, and they started using it on their own, and you just felt, ‘oh, this is so much fun.’ … Easy to use and nearby, … Then they (managers) moved it down to the entrance, and that ward hardly ever goes there, so you can see how the technology needs to be integrated, close to the staff and easy to use—then I really believe in it. – Interview 13

### Experiences of barriers in daily care that limit participation in quality improvement

This overarching theme integrates issues such as unclear role distribution, a sense of detachment from direct care work, and a weekly activity structure that limits health professionals’ participation in QI efforts. It also highlights the increased complexity of care. There is an emphasis on how structures and priorities distance health professionals from the core of care and reduce their ability to influence quality.

#### Unclear roles and distance from patient care hinder participating in quality improvement

The role descriptions for health professionals, along with job descriptions and the distribution of nursing responsibilities, were considered unclear. Health professionals felt disconnected from the care process and expressed a desire to engage more actively in enhancing care quality alongside nurse assistants and managers.

… there’s an awful lot of documentation, unfortunately. It feels like we’re getting further and further away from the patients. Before, we used to be on each ward, with the door slightly open, keeping track of how the patients were doing and what was going on, and it was easy for the staff to just pop their head in and ask something. I think it’s a bit sad, actually, that we’re getting further and further away. – Interview 15

The participants perceived that few improvement initiatives were taking place in the nursing homes, citing minimal or no examples of such efforts, which highlighted a general lack of QI initiatives. Additionally, the role of health professionals was only partially defined. While improving care quality is included in role descriptions, the practical implementation of these responsibilities remains ambiguous and has not been carried out systematically. The boundary between the responsibilities of unit managers and nurse assistants regarding social care and basic care, as well as the point at which healthcare begins, was poorly defined. The interplay between these two frameworks complicates a clear division of responsibilities, which the organization was structured to maintain. Both health professionals and nursing assistants tended to interpret their responsibilities differently. A lack of knowledge permeated the entire organization, from management to care staff, hindering QI efforts and the participation of health professionals.

Well, I’ve worked in the municipality before and felt that, sure, there’s always talk about change and improvement, but over time, you somehow always end up back at the same level. That’s how it feels. – Interview 1

#### The structure of incident management hinders participation in quality improvement

The approach to managing incidents did not promote participation. Health professionals indicated that they would like more feedback regarding actions taken and wish to participate in discussions on reducing incident statistics.

Many people are good at talking (managers). Uh … but you also notice that it doesn’t always lead to much when you raise something as a concern. It might get brought up at a staff meeting, but there’s no follow-up, and you don’t see that things get done as planned. – Interview 1

The current workflows and routines prevented health professionals from being involved in the follow-up of incidents and QI efforts related to them. This was largely because they were not included in the wards’ staff meetings due to the division between SoL and HSL. Issues were often discussed only during these meetings without any further follow-up. There was no assessment of what happened after the actions were implemented.

Well, one of the managers, at least, tends to be very defensive and focuses on defending the staff instead of recognizing where something could be improved. – Interview 12

A concern was an attitude in which managers defend existing practices that lead to incidents. The above quote suggests shortcomings in the safety culture.

… there are many incidents you can report, but then you feel like, well, you just don’t have the energy … – Interview 4

The lack of action on previously reported incidents has caused health professionals to lose trust in the system, leading them to perceive the documentation of incidents as an unnecessary burden. They felt that no meaningful changes or improvements were implemented to prevent recurrences, leading them to decide to not report all incidents they encountered.

#### The impact of increased care complexity on care quality and participation in quality improvement

Health professionals reported that care had become more complex at the nursing homes, with patients suffering from multiple chronic conditions requiring more advanced medical treatment. The increased care demands made it difficult to establish high-quality care and limited participation in QI efforts, as all professionals lacked time. Additionally, the development of care quality has not advanced and has not kept pace with the shift towards more complex and advanced care.

They are definitely more ill now than they have been in the past, and that brings its own challenges, both good and bad. There may not always be the resources for that, but … - Interview 10

A shortage of staff, coupled with untrained personnel lacking basic care education, was highlighted as a significant issue. Health professionals felt that the organization could not evolve or improve the quality of care due to insufficient time and knowledge. Additionally, a culture of “us vs them” seemed to prevail, with blame for poor care quality often directed at untrained healthcare workers and nursing assistants.

I’ve been around to a few different wards, and I can say that there are differences. And I believe it has a lot to do with the varying levels of competence among the staff on the floor. Then there’s also language. Language barriers. Sometimes I also feel that the staff working together in a group can’t really communicate with each other. – Interview 1

Health professionals expressed a desire to contribute more to improving care quality but felt hindered by the organizational division between SoL and HSL. Simultaneously, there was a pressing need for improvement due to the complexity of care. Health professionals were strongly motivated to advocate for change.

And what do you think is needed to make things better?

… Well, a safer and more secure care environment for the patients. Like, people should want to come to work and do a good job, have the competence for it, and the staffing to support it. And want to provide that extra quality. What you can clearly see is that a lot is being cut short in the care, maybe due to both lack of knowledge and lack of time, and we’re seeing the consequences of that, like a lot of fungal infections … those kinds of things. And then also just being able to offer some activities, doing something—maybe just helping someone read a newspaper, play a game, or sit and talk. That’s a huge gap. – Interview 10

Health professionals’ experiences have indicated that nursing homes have taken on more complex care responsibilities in recent years, at the same time there has been a diminished focus on QI initiatives. The frontline staff and management departments exhibited a lack of nursing competence and insufficient time for training, learning, and QI efforts. As a result, health professionals felt unable to take pride in the quality of care provided by the nursing homes, despite being responsible for it.

Do you feel proud of the care provided here by the team?

No. …. – Interview 4

## Discussion

The purpose of this study was to gain a deeper understanding of how health professionals in nursing homes experience participation in QI. The analysis highlights that health professionals encounter obstacles to both QI efforts and their participation in such initiatives. According to Organizational Readiness for Change theory (Weiner, 2009), these findings point to a low level of readiness for change within the organization. Numerous contextual factors, such as a lack of resources, hindering structures, and a resistant culture, complicate QI efforts in elderly care. Bergman *et al*. (2024) described how organizational barriers hinder development and QI in healthcare. Unlike the private sector, the public sector has not fully utilized the techniques and strategies of TQM, although doing so could be a potential remedy for the challenges it faces today. Health professionals leave healthcare because they perceive the quality of care is low, feel excluded from QI efforts, and believe that their expertise is not utilized (Gardulf *et al*., 2005).

Clear guidelines are essential to define who is responsible for QI and how health professionals can be engaged in the process. The lack of role clarity complicates efforts to implement new evidence and technologies in QI initiatives. This challenge was highlighted in a study in which middle managers in elderly care identified unclear role distribution as a significant obstacle to successful QI (Hartviksen *et al*., 2020). Additionally, upper management does not prioritize QI, which is evident in managers’ receiving training in scheduling but not in QI techniques (Hartviksen *et al*., 2020).

Structures to overcome these barriers are needed, along with an organizational framework in which QI is highly prioritized and valued by management, instead of the current short-term focus on budget and staffing issues. Furthermore, structures for fostering employee participation are lacking and must be developed. There are many techniques derived from TQM that can be utilized, including quality circles, problem-solving circles, and self-managed teams (Boaden, 1997). Most importantly, the organizational culture must shift to recognize employee participation as crucial for QI, a cornerstone of TQM (Bergman *et al*., 2022).

Breaking down the barriers that hinder QI between the SoL and HSL, ensuring that all professions are involved in QI, and driving a cultural shift from top management throughout the entire organization are critical steps described in the TQM literature for fostering a quality culture (Boaden, 1997). Given the increasing complexity of care and demographic changes, achieving this goal is essential for working long-term with QI and shifting the focus to meet the needs of patients and their families, a core concept within TQM (Boaden, 1997). A customer focus is deemed essential for improving quality, and this approach must be strengthened in the public sector. However, in healthcare, different terminology should be used (Bergman *et al*., 2024), and a person- or family-centred approach can serve as the foundation for QI.

The results of this study suggest that implementing an incident management system focused on the proactive prevention of incidents is crucial for QI. Such a system would foster a strong culture of patient safety within the organization. (Alabdaly *et al*., 2024). Cultural change, along with a shared understanding of why incidents are managed and the goals of incident management, is needed (Noghrehchi *et al*., 2024). To reduce conflicts and collaboratively enhance patient safety, the entire team must work together. Adjusting the work structure to support a patient safety culture and team-based QI can lead to several important outcomes. These include a shared commitment among team members (Guzzo and Dickson, 1996); a sense of ownership over the issues and contribution to organizational goals (Avey *et al*., 2009; Brown *et al*., 2014); a feeling of being an active part of the organization’s success (Knapp *et al*., 2014); and a shared understanding of team members’ purposes, roles, and responsibilities (Edwards *et al*., 2006). This presents an opportunity for health professionals to engage in systematically implementing evidence and new technology in QI efforts. By doing so, they can contribute more effectively to improving care processes and outcomes while fostering a culture of continuous improvement through strengthened empowerment of health professionals (Irwin *et al*., 2013). However, being reactive to incidents is not enough to drive comprehensive quality work. Listening to what staff identify as necessary improvements, alongside the needs and expectations of patients and their families, is the key to developing a structured QI process (Mohammad Mosadegh Rad, 2006). Research has shown that employee participation is the most critical core concept of TQM, serving as the key enabler for implementing other concepts of the theory within the organization (Bakotić and Rogošić, 2017).

Health professionals’ participation in QI may increase care quality in several ways: (1) Evidence-based practice and the implementation of new evidence aimed at improving care quality. If health professionals receive support and the necessary conditions to integrate evidence into their daily work, care quality will improve (Irwin *et al*., 2013). (2) Facilitating employee participation and gathering ideas from employees is described in TQM as essential for enhancing the quality of services provided (Bakotić and Rogošić, 2017). The current hierarchical structure in elderly care hinders this process however, if it is reversed, employees will have many ideas and suggestions for improvement, as shown in this study. TQM emphasizes that leadership engagement is needed to capture these ideas and create an innovative quality culture. (3) This study provides examples of employee-driven implementation of new technology, which yielded positive results. Research shows that employee participation is crucial for successful digital transformation in healthcare (Curtis and Brooks, 2020). (4) There is a strong desire among health professionals to work systematically with patient safety and incident management. If this engagement is harnessed, employee participation can drive QI. (5) Health professionals observe patient needs, such as increasing care complexity, and management must listen to them to make information-based decisions, which TQM identifies as critical for improving quality (Boaden, 1997).

### Implications for future research and limitations

This study indicates a general lack of systematic QI in nursing homes in Sweden, particularly with respect to the participation of health professionals in these efforts. This suggests the need for further research on how the involvement of health professionals in QI can be strengthened and facilitated within nursing homes and what is entailed in this context. Future studies should explore the conditions that enable participation in QI, exploring how leadership culture may need to change to foster such involvement. TQM tools need to be tested, evaluated, and adjusted to fit the context of elderly care.

The results cannot be considered representative of all nursing homes in Sweden. However, the findings provide important insights into how QI in elderly care and employee participation are perceived in nursing homes today. Further research is needed in this area to identify broader trends within this context.

## References

[ref001] Alabdaly, A., Hinchcliff, R., Debono, D. and Hor, S.-Y. (2024), “Relationship between patient safety culture and patient experience in hospital settings: a scoping review”, BMC Health Services Research, Vol. 24 No. 1, 906, doi: 10.1186/s12913-024-11329-w. 39113045 PMC11308681

[ref002] Avey, J.B., Avolio, B.J., Crossley, C.D. and Luthans, F. (2009), “Psychological ownership: theoretical extensions, measurement and relation to work outcomes”, Journal of Organizational Behavior, Vol. 30 No. 2, pp. 173-191, doi: 10.1002/job.583.

[ref003] Bakotić, D. and Rogošić, A. (2017), “Employee involvement as a key determinant of core quality management practices”, Total Quality Management and Business Excellence, Vol. 28 Nos 11-12, pp. 1209-1226, doi: 10.1080/14783363.2015.1094369.

[ref004] Bergman, B., Bäckström, I., Garvare, R. and Klefsjö, B. (2022), Quality from Customer Needs to Customer Satisfaction (4 Uppl), Studentlitteratur AB, Lund.

[ref005] Bergman, B., Klefsjö, B. and Sörqvist, L. (2024), “Swedish quality: a historical perspective and reflections for the future”, International Journal of Lean Six Sigma, Vol. 15 No. 6, pp. 1162-1186, doi: 10.1108/IJLSS-12-2023-0237.

[ref006] Boaden, R.J. (1997), “What is total quality management … and does it matter?”, Total Quality Management, Vol. 8 No. 4, pp. 153-172, doi: 10.1080/0954412979596.

[ref007] Braun, V. and Clarke, V. (2006), “Using thematic analysis in psychology”, Qualitative Research in Psychology, Vol. 3 No. 2, pp. 77-101, doi: 10.1191/1478088706qp063oa.

[ref008] Braun, V. and Clarke, V. (2019), “Reflecting on reflexive thematic analysis”, Qualitative Research in Sport, Exercise and Health, Vol. 11 No. 4, pp. 589-597, doi: 10.1080/2159676X.2019.1628806.

[ref009] Braun, V. and Clarke, V. (2021), “One size fits all? What counts as quality practice in (reflexive) thematic analysis?”, Qualitative Research in Psychology, Vol. 18 No. 3, pp. 328-352, doi: 10.1080/14780887.2020.1769238.

[ref010] Brown, G., Pierce, J.L. and Crossley, C. (2014), “Toward an understanding of the development of ownership feelings”, Journal of Organizational Behavior, Vol. 35 No. 3, pp. 318-338, doi: 10.1002/job.1869.

[ref011] Chamberlain, S.A., Hoben, M., Squires, J.E. and Estabrooks, C.A. (2016), “Individual and organizational predictors of health care aide job satisfaction in long term care”, BMC Health Services Research, Vol. 16 No. 1, 577, doi: 10.1186/s12913-016-1815-6. 27737672 PMC5064796

[ref012] Curtis, K. and Brooks, S. (2020), “Digital health technology: factors affecting implementation in nursing homes”, Nursing Older People, Vol. 32 No. 2, pp. 14-21, doi: 10.7748/nop.2020.e1236. 32159302

[ref013] Dahlgaard-Park, S., Chen, C.-K., Jang, J.-Y. and Dahlgaard, J.J. (2013), “Diagnosing and prognosticating the quality movement – a review on the 25 years quality literature (1987-2011)”, Total Quality Management and Business Excellence, Vol. 24 Nos 1/2, pp. 1-18, doi: 10.1080/14783363.2012.756749.

[ref014] Dahlgaard-Park, S.M., Reyes, L. and Chen, C.-K. (2018), “The evolution and convergence of total quality management and management theories”, Total Quality Management and Business Excellence, Vol. 29 Nos 9/10, pp. 1108-1128, doi: 10.1080/14783363.2018.1486556.

[ref015] Donabedian, A. (1985), “Twenty years of research on the quality of medical care: 1964-1984”, Evaluation and the Health Professions, Vol. 8 No. 3, pp. 243-265, doi: 10.1177/016327878500800301. 10301003

[ref016] Edwards, B.D., Day, E.A., Arthur, W. and Bell, S.T. (2006), “Relationships among team ability composition, team mental models, and team performance”, Journal of Applied Psychology, Vol. 91 No. 3, pp. 727-736, doi: 10.1037/0021-9010.91.3.727. 16737368

[ref017] Ersek, M. and Carpenter, J.G. (2013), “Geriatric palliative care in long-term care settings with a focus on nursing homes”, Journal of Palliative Medicine, Vol. 16 No. 10, pp. 1180-1187, doi: 10.1089/jpm.2013.9474. 23984636 PMC3996937

[ref018] European Commission (2018), “Challenges in long-term care in Europe”, available at: https://ec.europa.eu/social/main.jsp?langId=en&catId=89&newsId=9185

[ref019] Gardulf, A., Söderström, I., Orton, M., Eriksson, L.E., Arnetz, B. and Nordström, G. (2005), “Why do nurses at a university hospital want to quit their jobs?”, Journal of Nursing Management, Vol. 13 No. 4, pp. 329-337, doi: 10.1111/j.1365-2934.2005.00537.x. 15946172

[ref020] Guzzo, R.A. and Dickson, M.W. (1996), “Teams in organizations: recent research on performance and effectiveness”, Annual Review of Psychology, Vol. 47 No. 1996, pp. 307-338, doi: 10.1146/annurev.psych.47.1.307. 15012484

[ref021] Hackman, J.R. and Wageman, R. (1995), “Total quality management: empirical, conceptual, and practical issues”, Administrative Science Quarterly, Vol. 40 No. 2, pp. 309-342, doi: 10.2307/2393640.

[ref022] Hartviksen, T.A., Aspfors, J. and Uhrenfeldt, L. (2020), “Healthcare middle managers’ capacity and capability to quality improvement”, Leadership in Health Services, Vol. 33 No. 3, pp. 279-294, doi: 10.1108/LHS-11-2019-0072.

[ref023] ICN (2021), “The ICN Code of ethics for nurses”, available at: https://swenurse.se/nyheter-och-opinion/aktuellt/nyheter/2021-10-29-reviderad-icn-code-of-ethics-for-nurses

[ref024] Institute of Medicine (1990), US Committee to design a strategy for quality review and assurance in medicare. Medicare: A Strategy for Quality Assurance, Vol. 1 (Lohr, K.N., Ed.), National Academies Press, Washington, DC. PMID: 25144047, available at: http://www.ncbi.nlm.nih.gov/books/NBK235462/

[ref025] Irwin, M.M., Bergman, R.M. and Richards, R. (2013), “The experience of implementing evidence-based practice change: a qualitative analysis”, Clinical Journal of Oncology Nursing, Vol. 17 No. 5, pp. 544-549, doi: 10.1188/13.CJON.544-549. 24080054

[ref026] Klein, S., Brohm, K.-A. and Schadler, M. (2020), “The impact of understanding and organisational commitment on job involvement”, International Journal for Quality Research, Vol. 14 No. 4, pp. 1081-1096, doi: 10.24874/IJQR14.04-06.

[ref027] Knapp, M., Comas-Herrera, A., Wittenberg, R., Hu, B., King, D., Rehill, A. and Adelaja, B. (2014), “Scenarios of dementia care: what are the impacts on cost and quality of life?”, LSE Research Online Documents on Economics, available at: https://www.semanticscholar.org/paper/Scenarios-of-dementia-care%3A-what-are-the-impacts-on-Knapp-Comas-Herrera/c996a08b779a3f2de6f063810bf94f60de0706a8

[ref028] Kostman, J.T. and Sastry, S. (2019), “When implementing new technology, people are the key to success”, NACD Directorship, Vol. 45 No. 2, p. 61.

[ref029] Leung, P.P.L., Wu, C.H., Kwong, C.K., Ip, W.H. and Ching, W.K. (2021), “Digitalisation for optimising nursing staff demand modelling and scheduling in nursing homes”, Technological Forecasting and Social Change, Vol. 164, 120512, doi: 10.1016/j.techfore.2020.120512.

[ref030] Mid Sweden University (2021), “Mid Sweden university quality assurance for research”, available at: https://www.miun.se/medarbetare/forskning/styrning-och-etik/forskningsetik-forskare-forskarstuderande/

[ref031] Mohammad Mosadegh Rad, A. (2006), “The impact of organizational culture on the successful implementation of total quality management”, The TQM Magazine, Vol. 18 No. 6, pp. 606-625, doi: 10.1108/09544780610707101.

[ref032] National Board of Health and Welfare (2016), Your Right to Care and Nursing. A Guide for the Elderly [Din Rätt till Vård Och Omsorg. En Vägvisare För Äldre ], Socialstyrelsen, Stockholm.

[ref033] Noghrehchi, P., Hefner, J.L. and Walker, D.M. (2024), “The relationship between hospital patient safety culture and performance on Centers for Medicare and Medicaid Services value-based purchasing metrics”, Health Care Management Review, Vol. 49 No. 4, pp. 281-290, doi: 10.1097/HMR.0000000000000414. 39104010

[ref034] Patton, M.Q. (2015), Qualitative Research and Evaluation Methods: Integrating Theory and Practice, SAGE Publications, Saint Paul, MN.

[ref035] SFS (2001), SFS 2001:453: Social Services Act, Ministry of Health and Social Afairs, Stockholm.

[ref036] SFS (2017), SFS 2017:30: Health and Medical Services Act, Ministry of Health and Social Afairs, Stockholm.

[ref037] Swedish Research Council (2017), “Good Research Practice”, available at: https://www.vr.se/english/analysis/reports/our-reports/2017-08-31-good-research-practice.html

[ref038] Weiner, B.J. (2009), “A theory of organizational readiness for change”, Implementation Science, Vol. 4 No. 1, p. 67, doi: 10.1186/1748-5908-4-67. 19840381 PMC2770024

[ref039] Werner, R.M., Konetzka, R.T. and Kim, M. (2013), “Quality improvement under nursing home compare: the association between changes in process and outcome measures”, Medical Care, Vol. 51 No. 7, pp. 582-588, doi: 10.1097/MLR.0b013e31828dbae4. 23756645 PMC3683894

[ref040] Winters, S., Cornelissen, M.M.E., Kool, T., Klazinga, N. and Huijsman, R. (2016), “How do Dutch residential care homes and home care organizations improve their qualtiy? A description of quality improvement activities and the influence of organization characteristics on quality improvement”, International Journal for Quality in Health Care, Vol. 26 No. 4, doi: 10.13140/RG.2.1.1231.5285. 24872324

[ref041] Zink, K.J. (2008), “Human resources and organisational excellence”, Total Quality Management and Business Excellence, Vol. 19 Nos 7-8, pp. 793-805, doi: 10.1080/14783360802159451.

[ref042] Zuidgeest, M., Strating, M., Luijkx, K., Westert, G. and Delnoij, D. (2014), “Using client experiences for quality improvement in long-term care organizations”, International Journal for Quality in Health Care, Vol. 26 No. 5, p. 571, doi: 10.1093/intqhc/mzs041. 22490298

